# D. G. Westcott

**Published:** 1846-12

**Authors:** 


*Obituary.*—D. G. Westcott, M. D., an eminent dental surgeon, of this city, died in Port Gibson, on Tuesday, the 22d inst., from hæmorrhage of the lungs. The Doctor attended church on the Sabbath previous, and communed in the celebration of the Lord's supper. On Tuesday, immediately after extracting a tooth for a lady, he was seized with a profuse hæmorrhage, and expired in a quarter of an hour. The deceased was an amiable and pious man; kind, unobtrusive and benevolent; and of great eminence and usefulness in his profession. Though he died from home, and far away from his wife, who is on a visit to her relations in New York, yet he died in the midst of a large circle of friends, endeared to him by many ties, and who paid every attention and respect to his remains that the solemn occasion demanded.—*Jackson Southron.*

